# Molecular identification and antifungal susceptibility testing of *Candida* species isolated from oral lesions in patients with head and neck cancer undergoing radiotherapy

**DOI:** 10.18502/cmm.7.1.6242

**Published:** 2021-03

**Authors:** Firoozeh Kermani, Mohaddese Sadeghian, Tahereh Shokohi, Seyedebrahim Hashemi, Dariush Moslemi, Saeed Davodian, Mahdi Abastabar, Zainab Bandalizadeh, Leyla Faeli, Zahra Seifi, Mahmoud Fami Zaghrami, Iman Haghani

**Affiliations:** 1 Student Research Committee, School of Medicine, Mazandaran University of Medical Sciences, Sari, Iran; 2 Invasive Fungi Research Center, Communicable Diseases Institute, Mazandaran University of Medical Sciences, Sari, Iran; 3 Department of Medical Mycology, School of Medicine, Mazandaran University of Medical Sciences, Sari, Iran; 4 Department of Radiology and Radiation Therapy, School of Medicine, Babol University of Medical Sciences, Babol, Iran; 5 Department of Radiation Oncology, School of Medicine, Imam Khomeini Hospital, Mazandaran University of Medical Science, Sari, Iran; 6 Department of Microbiology, School of Veterinary Medicine, Islamic Azad University, Babol, Iran

**Keywords:** Antifungal susceptibility test, *Candida* species, Head and neck cancer, Oropharyngeal candidiasis, Radiotherapy

## Abstract

**Background and Purpose:**

Radiation therapy in patients with head and neck malignancies predisposes them to oral *Candida* colonization and infection due to damage of oral mucosa and destruction of the salivary gland. This study aimed to determine the prevalence of oropharyngeal candidiasis (OPC) in patients with head and neck cancer (HNC) undergoing radiotherapy (RT), identify the yeasts isolated from them, and determine their antifungal susceptibility.

**Materials and Methods:**

This cross-sectional study was conducted from December 2018 to June 2019 at two referral radiotherapy centers in northern Iran. Yeast strains that were isolated from patients with HNC were identified using conventional and molecular methods. The *in vitro* activities of eight common antifungal drugs against 55 isolates were investigated according to the guidelines of the Clinical and Laboratory Standard Institute (M27-A3 and M27-S4) broth microdilution document.

**Results:**

Among 59 patients receiving RT, the prevalence of OPC was 21 (35.59%) and 15 (25.42%) patients were diagnosed with colonization. The mean age of the patients was 55.32±13.3 years (within the range of 27-87 years). In this study, the pseudomembranous form was reported as the most clinical type of OPC. *Candida albicans* with the frequency of 60% was the most common type of *Candida* spp. that was observed in this study, although non-*albicans* *Candida* spp., such as *C. glabrata* (27.27%), *C. tropicalis* (5.45%), *C. parapsilosis* (3.63%), *C. krusei* (1.83%), and *C. kefyr* (1.83%) were also isolated. Considering the low minimum inhibitory concentration range of amphotericin B, compared to fluconazole, administration of this agent is a more suitable antifungal drug for extensive oral candidiasis in these patients. Among azoles, clotrimazole had low efficacy and several studied isolates (65.5%) showed resistance.

**Conclusion:**

Correct diagnosis as well as determining drug sensitivity and risk factors are the effective steps in reducing the complications related to oral candidiasis in people undergoing RT.

## Introduction

Patients undergoing radiation therapy (RT) for head and neck cancer (HNC) experience confluent mucositis three weeks after starting the therapy due to mucosal atrophy and decreased epithelial cell regeneration. Saliva provides a local mucosal defense that may sweep bacteria and yeast on swallowing. Hypo-salivation, mucositis, cancer radiation, and chemotherapy are factors that lead to the overgrowth of commensal yeasts and their shift to the pathogen [ 1 , 2 ]. 

Oropharyngeal candidiasis (OPC) is an opportunistic fungal infection caused by the overgrowth of *Candida* species in the mouth and throat that is also known as thrush [ 2 , 3 ]. *Candida* spp. are one of the normal oral flora in mucous membranes [ 4 ]. Imbalance of oral microbiota causes *Candida* to multiply; accordingly, the person’s own flora can be the main cause of the OPC. Many predisposing factors result in changing the status of the benign colonization to a pathological state that has severe local or serious systemic consequences [ 5 , 6 ].

Worldwide, HNC accounts for more than 650,000 cases and 330,000 deaths annually. The most important underlying conditions for OPC are uncontrolled diabetes mellitus, antimicrobial therapy, oral or inhaled corticosteroid usage, ill-fitting dentures, xerostomia, chemotherapy for neoplastic disease, RT of HNC [ 7 , 5 ]. The OPC is a major cause of morbidity in patients with HNC undergoing RT that may exacerbate oral mucositis [ 8 , 9 ]. 

In a retrospective cohort study, OPC and *Candida* colonization were reported in 37% and 70% of patients undergoing concurrent chemo-radiotherapy for HNC, respectively [ 5 ]. In another study, a higher rate of oral *Candida* colonization (up to 93%) and in like manner OPC (up to 30%) were observed in patients with oral cancer undergoing RT [ 10 ]. Usually, OPC is associated with the development of oral pain or burning and dysgeusia (taste changes), anorexia, malnutrition, and dysphagia in the RT [ 9 ]. 

Various *Candida* spp., including *C. albicans*, *C. tropicalis*, *C. krusei*, *C. parapsilosis*, *C. glabrata*, *C. guilliermondii*, *C. parapsilosis*, *C. kefyr*, *C. dubliniensis*, *C. viswnathii*, and *C. stellatoidea*, were isolated from the patients with HNC undergoing RT [ 11 , 12 ]. Until recently, *Candida albicans*, as the most common agent, has been responsible for 80–90% of OPC. Its prevalence might have been related to its greater adherence to epithelial cells for establishing oral colonization and infection, compared to non-*albicans* *Candida* spp. [ 13 , 5 ]. Moreover, it might be associated with other *Candida* virulence and the host factors [ 10 ]. 

In recent years, there has been a significant shift from *C. albicans* toward non-*albicans* *Candida* spp. as the common causative agents [ 14 , 15 ]. The non-*albicans* *Candida* spp. are more resistant to azole antifungal agents and represent a major clinical and public health challenge [ 16 ]. The present study aimed to investigate the prevalence of OPC in patients with HNC undergoing RT and determine the *in vitro* antifungal susceptibilities of eight common antifungal agents against *Candida* species.

## Materials and Methods

This cross-sectional study was conducted during a six-month period (from December 2018 to June 2019) at two referral radiotherapy centers of Shahid Rajaee Hospital in Babolsar, and Imam Khomeini Hospital in Sari, northern Iran. This study was approved by the Ethics Committee of Mazandaran University of Medical Sciences, Sari, Iran (IR.MAZUMS.REC.96.3006) and performed in compliance with the Declaration of Helsinki. Written informed consent was obtained from the patients and legal guardians regarding the inclusion of details in the manuscript and their publication.

In total, 59 patients with HNC including thyroid, oral cavity (hard palate, soft palate, and tongue), hypopharynx, larynx, and lymphoma undergoing the course of RT were enrolled in a sequential manner according to the clinical criteria of OPC, including complaints of burning, loss of taste, mucositis, eating or swallowing difficulties (dysphagia) associated with redness, and symptoms of white patches (plaques) on the inner cheeks, tongue, and palates. The patients with recent use of antifungal drugs were excluded from the study. The demographic data of the participants, such as age, gender, and type of cancer, predisposing factors (i.e., denture usage, smoking habits, previous antibiotic and chemotherapeutic agents usage ), comorbidities with an increased risk of OPC (including diabetes), and clinical features were recorded in their data sheets. 

Their oral hygiene was assessed using the Simplified Oral Hygiene Index (OHI-S) by summing up the Sampling the Debris Index-Simplified and Calculus Index-Simplified. Based on the results of the OHI-S assessment, the subjects were categorized as good (0-1.2), fair (1.3-3.0), and poor (3.1-6.0) as previously described [ 17 ]. 

It should be mentioned that sampling was carried out using sterile saline wetted swabs. Patients with verified OPC were selected based on conventional methods for the detection of budding yeast cells and/or pseudohyphae in KOH (10%) mount, yeast growth on Sabouraud dextrose agar (SDA) (Merck, Germany), SDA supplemented with 0.5% chloramphenicol and CHROMagar *Candida* (CHROMagar Company, Paris, France) media incubated at 35 °C for 48-72 h. The chromogenic medium was used for presumptive identification of mixed yeast spp. in a specimen. The extent of growth was assessed semiquantitatively and categorized as none, light (<10 colonies), moderate (10-100 colonies), or heavy (>100 colonies) [ 18 ]. Negative direct microscopy result together with a small number of yeasts (<10 colonies) was considered as *Candida* colonization rather than infection.

For molecular identification, genomic DNA was extracted according to the previously described method and the amplification of DNA was performed using universal primers (ITS1 and ITS4), in a total reaction volume of 25 µl. Afterward, the restriction enzyme examination was performed with restriction enzyme *Msp1* (Fisher Scientific, Leicestershire, UK) as previously described [ 19 ].

Broth microdilution antifungal susceptibility testing of eight antifungals, including amphotericin B (AMB), nystatin (NYS), voriconazole (VOR), itraconazole (ITR), miconazole (MIC), clotrimazole (CLO), and ketoconazole (KET) (0.016-16 µg/ml) as well as fluconazole (FLC) (0.063-64 µg/ml) against the isolates were determined using the Clinical and Laboratory Standards Institute M27-A3 and M27-S4 document guidelines [ 20 ]. 

All the drugs were obtained from Sigma-Aldrich Company (Sigma-Aldrich, Steinheim, Germany) and dissolved in 1% dimethyl sulfoxide (Sigma). Briefly, fresh and mature colonies were suspended in sterile water, and the optical density at 530 nm was adjusted to the 75-77% transmission range of 0.5-2.5×10^3^ cells/ml. Plates were incubated at 35 °C and investigated after 24 h. The minimum inhibitory concentration (MIC) endpoint was defined as 100% inhibition for AMB and NYS and 50% inhibition for the other drugs. It should be noted that all the isolates were tested in duplicate. The quality control strains were *C. krusei* ATCC 6258 *C. parapsilosis* ATCC 22019.

Statistical analysis

The collected data were analyzed in SPSS software (version 16, Statistical Product and Services Solutions, Inc, Chicago, IL, USA), and a *p*-value of ≤ 0.05 was considered statistically significant.

## Results

This study was conducted on 59 HNC patients undergoing RT. Based on the findings, 36 (61.01%) out of 59 patients had a positive culture for *Candida* spp. Among the patients with a positive culture, 21 (58.33%) and 15 (41.66%) cases were diagnosed with OPC and *Candida* colonization, respectively (Table 1). The patients were within the age range of 27-87 years and their mean age was 55.32±13.3 years. Moreover, 36 (61%) patients were male and 23 (39 %) were female. 

**Table1:** Demographic and clinical characteristics of 59 patients with head and neck cancer undergoing radiography and distribution of patients based on oropharyngeal candidiasis or oral colonization

Type of *Candida* Involvement	Gender		Diabetic	Tobacco use	Dental prosthesis	Oral health status*			Previous broad-spectrum antibacterial usage	Chemotherapy	Radiation duration		Radiation dose**	
	Female	male				P	F	G			<25 sessions	>25sessions	<5500 cGY	>5500 cGY
OPC*** 21/59 (35.59%)	8	13	1	4	8	2	12	7	3	9	12	9	14	7
Oral COL**** 15/59 (25.42%)	7	8	2	5	6	1	11	3	1	7	11	4	11	4
Total *Candida* involvement (%)	15/36 (41.66%)	21/36(58.3%)	3/36 (8.33%)	9/36 (25%)	14/36 (38.9%)	3/36 (8.33%)	23/36 (63.9%)	10/36 (27.7%)	4/36 (11.1%)	16/36 (44.44%)	23/36 (63.9%)	13/36 (36.11%)	25/36 (69.44%)	11/36 (30.55%)
Total (%)	23/59 (38.9%)	36/59 (61.01%)	6/59 (10.16%)	13/59 (22.0%)	20/59 (33.89%)	5/59 (8.47%)	29/59 (49.15%)	25/59 (42.37%)	7/59 (11.86%)	26/59 (44.06%)	38/59 (64.40%)	21/59 (35.59%)	45/59 (76.2%)	14/59 (23.72%)

Note: Oral Health status

* : P=poor; F=Fair, G=good; Radiation dose

** : cGy= centigray, it is 0.01 of a single gray unit, OPC

*** : oropharyngeal candidiasis, COL

**** : colonization

In the present study, the most common clinical feature was pseudomembranous plaques (n=13, 61.9%), erythematous (n=5, 23.8%), and leukoplakia (n=3, 14.28%). The patients often complained of burning and dysphagia. In addition, 13 (22%) patients had a history of smoking, and diabetes was reported in only 6 (10.2%) patients. Besides, 20 (33.9%) participants had a history of wearing dentures and their oral hygiene was assessed at three levels of poor (n=4, 6.8%), fair (n=13, 32.2%), and good (n=36, 61%). A significance level was estimated for the relationship between oral health statuses and OPC of people undergoing radiotherapy (*p*-value≤0.001). The results revealed that there was no significant relationship between chemotherapy with OPC in patients receiving the combined chemotherapy and RT (*p*-value=0.85).

In this study, 55 strains of *Candida* species were identified in 36 patients as causative agents using polymerase chain reaction-restriction fragment length polymorphism (PCR-RFLP). The *C. albicans* was the predominant spp. (n=33; 60%) followed by *C. glabrata* (n=15; 27.3%), *C. tropicalis* (n=3; 5.5%), *C. parapsilosis* (n=2; 3.6%), *C. krusei* (n=1; 1.8 %), and *C. kefyr* (n=1; 1.8 %) (Table 2). In 22% (n=13) of the patients, multiple *Candida* spp. were observed as causative agents, and in most of them (69.23%; 9/13), *C. albicans* were mixed with *C. glabrata* (Table 2). 

**Table2:** Distribution of *Candida* spp. (as single or multiple) isolated from head and neck cancer patients undergoing radiotherapy with oropharyngeal candidiasis or oral colonization

*Candida* species		Oral *Candida* colonization no. (%)	Oropharyngeal candidiasis no. (%)	Total no. (%)
Single species	*C. albicans*	4 (44.4)	29 (63)	33 (60)
	*C. glabrata*	3 (33.3)	12 (26)	15 (27.27)
	*C. tropicalis*	0	3 (6.5)	3 (5.45)
	*C. parapsilosis*	0	2 (4.3)	2 (3.63)
	*C. kefyr*	1 (11.1)	0	1 (1.81)
	*C. krusei*	1 (11.1)	0	1 (1.81)
	Total species	9 (16.36)	46 (83.63)	55 (100)
Multiple species	*C. albicans-C. glabrata*	5 (38.46)	4 (30.76)	9 (69.23)
	*C. albicans-C. glabrata- C. kefyr*	1 (7.69)	-	1 (7.69)
	*C. albicans-C. parapsilosis*	-	1 (7.69)	1 (7.69)
	*C. albicans-C. tropicalis*	-	1 (7.69)	1 (7.69)
	*C. tropicalis-C. parapsilosis*	-	1 (7.69)	1 (7.69)
	Total cases	6 (46.15)	7 (53.84)	13 (100)

Analysis of the data showed that there was no significant relationship between the frequency of OPC or ‎colonization and the incidence of multi-species (*p*-value=0.71). Table 3 shows the *in vitro* susceptibility testing results (MIC_50_, MIC_90_, geometric mean (GM), and MIC ranges) for 55 clinical isolates of *Candida* spp. that were isolated from patients with HNC. Among azole antifungals, CLO had the highest GM MIC (2.38 µg/ml) and MIC_50_ (4 µg/ml). The lowest MIC_50_ value (0.032 μg/mL) against *C. albicans*, *C. glabrata*, and *C. tropicalis* belonged to VOR and KET. Moreover, *C. parapsilosis* spp. also had a low MIC range (0.125-0.5 µg/ml) and MIC_50_ value (0.35 µg/ml) against voriconazole (Table 3). 

**Table3:** Results of susceptibility pattern of *Candida* species isolated from head and neck cancer patients undergoing radiotherapy against eight antifungals

*Candida* species	Antifungal agents*	0.016	0.032	0.063	0.125	0.25	0.5	1	2	4	8	16	32	≥64	MIC Range (µg/mL)	MIC_50_ (µg/mL)	MIC_90_ (µg/mL)	G-Mean (µg/mL)	Mode (µg/mL)
*C. albicans*(n=33)	AMB	-	1	-	2	1	8	19	2	-	-	-	-	-	0.032-2	1	1	0.67	1
	NYS	-	-	-	-	-	1	12	14	5	1	-	-	-	0.5-8	2	4	1.72	2
	ITR	1	5	10	6	2	2	1	-	2	1	3	-	-	0.016-16	0.125	7.2	0.204	0.063
	FLC	-	12	2	6	3	4	4	-	1	-	-	-	1	0.032-64	0.125	1	0.158	0.032
	VOR	5	14	6	-	-	-	-	1	1	-	6	-	-	0.016-16	0.032	16	0.132	0.032
	KET	7	14	2	1	1	1	-	-	1	2	4	-	-	0.016-16	0.032	14.4	0.119	0.032
	MIC	-	9	5	3	5	4	1	-	1	1	4	-	-	0.032-16	0.125	14.4	0.246	0.032
	CLO	-	1	3	4	3	1	2	-	2	1	16	-	-	0.032-16	8	16	2.08	16
*C. glabrata*(n=15)	AMB	-	-	-	-	-	1	11	3	-	-	-	-	-	0.5-2	1	2	1.09	1
	NYS	-	-	-	-	-	-	6	7	2	-	-	-	-	1-4	2	3.2	1.66	2
	ITR	-	3	1	2	2	5	2	-	-	-	-	-	-	0.032-1	0.25	0.8	0.20	0.5
	FLC	-	4	-	4	1	-	-	4	2	-	-	-	-	0.032-4	0.125	3.2	0.30	0.032
	VOR	-	11	4	-	-	-	-	-	-	-	-	-	-	0.032-0.063	0.032	0.063	0.038	0.032
	KET	-	10	2	1	2	-	-	-	-	-	-	-	-	0.032-0.25	0032	0.2	0.05	0.032
	MIC	-	3	-	1	1	2	2	3	1	1	1	-	-	0.125-16	1	6.4	0.63	0.032
	CLO	-	-	-	1	-	-	2	3	1	1	7	-	-	0.125-16	8	16	4.59	16
*C. parapsilosis* (n=2)	AMB	-	-	-	-	-	-	2	-	-	-	-	-	-	1	1	ND	1	1
	NYS	-	-	-	-	-	-	2	-	-	-	-	-	-	1	1	ND	1	1
	ITR	-	-	-	-	-	2	-	-	-	-	-	-	-	0.5	0.5	ND	0.5	0.5
	FLC	-	-	-	-	-	-	-	-	-	1	1	-	-	8-16	11.31	ND	11.31	ND
	VOR	-	-	-	1	-	-	1	-	-	-	-	-	-	0.125-1	0.35	ND	0.35	ND
	KET	-	-	-	-	-	2	-	-	-	-	-	-	-	0.5	0.5	ND	0.5	0.5
	MIC	-	-	-	-	-	-	-	2	-	-	-	-	-	2	2	ND	2	2
	CLO	-	-	-	-	-	-	-	-	2	-	-	-	-	4	4	ND	4	4
*C. tropicalis*(n=3 )	AMB	-	-	1	1	-	-	-	1	-	-	-	-	-	0.063-2	0.125	ND	0.25	ND
	NYS	-	-	-	-	-	1	1	-	1	-	-	-	-	0.5-4	1	ND	1.26	ND
	ITR	-	-	1	1	-	-	-	-	-	-	1	-	-	0.063-16	0.125	ND	0.5	ND
	FLC	-	1	-	1	-	1	-	-	-	-	-	-	-	0.032-0.5	0.125	ND	0.157	ND
	VOR	1	1	-	-	-	-	-	-	-	1	-	-	-	0.016-8	0.032	ND	0.16	ND
	KET	1	1	-	-	-	-	-	-	-	1	-	-	-	0.016-8	0.032	ND	0.16	ND
	MIC	-	1	-	-	-	-	1	-	-	-	1	-	-	0.032-16	0.8	ND	0.8	ND
	CLO	-	1	-	-	-	-	1	-	-	-	1	-	-	0.032-16	0.8	ND	0.8	ND
*C. kefyr*(n=1)	AMB	-	-	-	-	-	-	-	1	-	-	-	-	-	2	ND	ND	ND	ND
	NYS	-	-	-	-	-	-	-	-	-	1	-	-	-	8	ND	ND	ND	ND
	ITR	-	-	-	-	1	-	-	-	-	-	-	-	-	0.25	ND	ND	ND	ND
	FLC	-	-	-	1	-	-	-	-	-	-	-	-	-	0.125	ND	ND	ND	ND
	VOR	1	-	-	-	-	-	-	-	-	-	-	-	-	0.016	ND	ND	ND	ND
	KET	1	-	-	-	-	-	-	-	-	-	-	-	-	0.016	ND	ND	ND	ND
	MIC	-	-	-	-	-	1	-	-	-	-	-	-	-	0.5	ND	ND	ND	ND
	CLO	-	-	-	-	-	1	-	-	-	-	-	-	-	0.5	ND	ND	ND	ND
*C. krusei*(n=1)	AMB	-	-	-	-	-	1	-	-	-	-	-	-	-	0.5	ND	ND	ND	ND
	NYS	-	-	-	-	-	-	-	1	-	-	-	-	-	2	ND	ND	ND	ND
	ITR	-	-	-	-	-	-	1	-	-	-	-	-	-	1	ND	ND	ND	ND
	FLC	-	1	-	-	-	-	-	-	-	-	-	-	-	0.032	ND	ND	ND	ND
	VOR	-	-	-	-	1	-	-	-	-	-	-	-	-	0.25	ND	ND	ND	ND
	KET	-	-	-	-	-	-	-	1	-	-	-	-	-	2	ND	ND	ND	ND
	MIC	-	-	-	-	-	-	-	-	-	-	1	-	-	16	ND	ND	ND	ND
	CLO	-	-	-	-	-	-	-	-	-	-	1	-	-	16	ND	ND	ND	ND
All *Candida* species (n=55)	AMB	-	1	1	3	1	10	32	7	-	-	-	-	-	0.032-2	1	2	0.77	1
	NYS	-	-	-	-	-	2	21	22	8	2	-	-	-	0.5-8	2	4	1.7	2
	ITR	1	8	12	9	5	9	3	-	2	2	4	-	-	0.016-16	0.125	4	0.22	0.063
	FLC	-	18	2	12	4	5	4	4	3	1	1	-	1	0.032-64	0.125	3.2	0.21	0.031
	VOR	7	26	10	1	1	-	-	2	1	1	6	-	-	0.016-16	0.031	12.8	0.09	0.031
	KET	9	25	4	2	3	3	-	1	1	3	4	-	-	0.016-16	0.031	8	0.099	0.031
	MIC	-	13	5	4	6	7	4	5	2	2	7	-	-	0.032-16	0.25	12.8	0.39	0.031
	CLO	-	2	3	5	3	2	4	3	5	3	25	-	-	0.032-16	4	16	2.38	16	

Note: Antifungal agents

* : FLC=fluconazole, AMB=amphotericin B, KET=ketoconazole, ITR=itraconazole, VOR=voriconazole, MIC=miconazole, CLO=clotrimazole, NYS=nystatin, ND= not determined

## Discussion

The OPC is one of the most serious problems for patients with HNC undergoing RT [ 9 ]. In a retrospective cohort study, OPC and *Candida* colonization were reported in 37% and 70% of patients with HNC undergoing chemo-radiotherapy, respectively [ 5 ]. In another study, the results indicated a higher rate of oral *Candida* colonization (up to 93%) and in like manner OPC (up to 30%) in patients with oral cancer undergoing RT [ 10 ]. Usually, OPC is associated with the development of oral pain or burning and dysgeusia (taste changes), anorexia, malnutrition, and dysphagia in radiation therapy [ 9 ]. 

Lack of diagnosis and differentiation between oral mucositis and oral candidiasis due to their clinical similarities poses major challenges to the treatment of these patients and can lead to their exacerbations [ 12 ]. Due to the emergence of resistance in *Candida* spp., it is necessary to accurately and timely identify the *Candida* spp. causing OPC using molecular techniques and determination of their susceptibility against the antifungal agents. This identification will help to choose the proper medication in the early stages of the disease, provide appropriate management, and consequently, reduce exacerbations.

Both structural and functional changes of salivary glands caused by RT play important roles in the development of OPC and shift of the growth of *Candida* as an oral commensal organism to a pathogenic one [ 21 ]. In the present study, 36 out of 59 patients with HNC undergoing RT were confirmed to have OPC or colonization. It is worth mentioning that the majority of these cases were male. This is in line with the findings of previous studies in the related literature [ 8 , 11 ].

The chemo-radiotherapy leads to an increase in the prevalence of OPC with irritation of the oral mucosa in patients with HNC [ 22 - 24 ]. Based on the results of this study, nine and seven cases out of the total number of patients receiving both chemotherapy and RT were reported to have OPC and colonization, respectively. The data analysis revealed that there was no significant relationship between total colonization and oral candidiasis in patients receiving the combined chemotherapy and RT which is inconsistent with the results of a study performed by Bashir et al. [ 25 ]. During the last decade, there have been many reports about the increase in the prevalence of non-*albicans* *Candida* spp. in such patients [ 8 , 26 , 27 ]; however, *C. albicans* is still the cause of most of the OPC [ 11 , 12 , 28 ].

In this study, based on both traditional and molecular methods, *C. albicans* was found to be the most causative agent. At this point, the PCR-RFLP method was used in all samples for the identification of yeast spp. along with microscopic observation methods and culture and biochemical tests [ 28 ]. The predominant non-albicans *Candida* spp. was *C. glabrata* followed by *C. tropicalis*, *C. parapsilosis*, *C. kefyr*, and *C. krusei*, which is consistent with previous studies [ 11 , 29 , 22 ]. In a previous study, the prevalence rate of oral infection due to uncommon yeasts was 6.1%, while in the present study, no cases of unusual yeast agents were found [ 30 ].

In 22% of cases, more than one *Candida* spp. were isolated using CHROMagar *Candida* medium; they were considered as patients with candidiasis caused by multiple spp. The *C. glabrata*, *C. tropicalis*, and *C. parapsilosis* may be the emerging causes of oropharyngeal infection [ 8 ]; however, the pathogenic role of these species in the absence of co-infection with *C. albicans* remains controversial [ 9 ]. 

In this study, no significant relationship was found between the frequency of OPC or colonization and the incidence of multi-species infection. Redding et al. have suggested that mixed infection of *C. albicans* with *C. glabrata* often requires high doses of FLC for treatment. It was noted that the previous FLC usage might cause the emergence of *C. glabrata*, and as mentioned, detection of the causative agents at the spp. level with more accurate techniques can lead to successful treatment [ 8 ]. Therefore, management of the above-mentioned associated factors can be effective in decreasing the treatment failure in the patients receiving RT. 

In this study, risk factors, such as tobacco use, antibiotic use, oral hygiene, and the use of denture prostheses were investigated in HNC patients undergoing RT. The results showed that 49% of cases had moderate oral hygiene and the prevalence of OPC and colonization was higher in them, compared to others (63.9%). Based on the results of a previous study, FLC prophylaxis was effective on severe mucositis in patients undergoing RT and chemotherapy [ 31 ]. Nevertheless, the emergence of antifungal-resistant species and the increase of the oral colonization by non-*albicans* *Candida* strains are among the RT and chemo-radiotherapy consequences [ 32 , 9 ]. 

According to the findings, the MIC_50_ value was 0.125 µg/ml for all of the isolated *C. albicans* against FLC except for one isolate from a patient with a history of diabetes whose MIC was ≥ 16 µg/ml. This is consistent with the results of the studies conducted by Yogitha et al. and Mohd Suhail Lone et al. [ 29 , 33 ]. Moreover, in the present study, two isolates of *C. parapsilosis* had a MIC of ≥ 8 µg/ml against FLC. Resistance to CLO was observed in 36 (65.5%) of the studied isolates which is consistent with the findings of a previous study [ 34 ]. 

In molecular identification of the isolates in this study, only one *C. kefyr* was detected as a non-*albicans* *Candida* spp. colonizer which was sensitive to all the studied azoles. In this regard, Jahanshiri et al. reported three isolates of *C. kefyr* in OPC of HNC patients, and one of them was truly sensitive to FLC and ITR [ 11 ]. The resistance rate of *Candida* isolates against ITR and KET was 20.0% in this study. It must be noted that this rate varied in different studies [ 21 , 35 , 11 ]; for instance, in a study, *Candida* isolates showed 7.7% and 4.7% resistance to ITR and KET, respectively, which are lower than the rates found in the present study [ 22 ]. 

In this study, the MIC_90_, GM, and resistance rate of NYS against all *Candida* spp. were 4 µg/ml, 1.7 µg/ml, and 18.18%, respectively. Katiraee et al. [ 34 ] and Yogitha et al. [ 29 ] reported that all *Candida* isolates were susceptible to NYS which is common among people with oral lesions. However, this is in contrast with the findings of another study that indicated resistance of *Candida* isolates against NYS [ 36 ]. 

In the present study, all of the *Candida* strains of cancer patients exhibited low MIC values to AMB with a GM of 0.77 μg/ml which is inconsistent with the results of previous studies that reported high resistance to AMB [ 37 , 34 ]. However, it is similar to the findings of some of the previous studies that indicated most isolates of *C. albicans* were sensitive to AMB [ 38 , 34 ]. According to the results of the present research, two *C. parapsilosis* and one *C. albicans* isolates that have a high MIC range against FLC had a good response to AMB. Therefore, it is recommended to use AMB in the treatment of FLC-resistant candidiasis in cancer patients.

This research was limited by the small size of the study population due to the strict enrollment. The patients who were using over-the-counter azole antifungals due to an annoying and painful oral lesion were often excluded from the study. However, these findings might still be applicable in the management of OPC in HNC patients undergoing RT due to the paucity of related studies in the current literature.

## Conclusion

The *C. albicans* are the most causative agent of OPC infection in HNC patients (60%) and *C. glabrata* is the predominant non-*albicans* *Candida* spp. (40%). Given the lower MIC range of AMB, compared to FLC, administration of this agent is more suitable for oral candidiasis in HNC patients. Among azoles, CLO had low efficacy and 65.5% of the studied isolates showed resistance against it. Management of risk factors, determination of the causative agent at the spp. level, and identification of the pattern of antifungal susceptibility can play important roles in the reduction of the morbidity of OPC and exacerbation of the oral mucositis in patients undergoing RT.

## Authors’ contribution

T. S., M. S., D.M., S.D., and M.A. conceived, designed, and coordinated the research. M.S., S.H., F.K., Z.B., L.F., Z.S., M.F.Z., and I. H. collected the data. T. S. and F.K. wrote the paper. All authors revised the manuscript and contributed to the improvement of the paper. All authors read and approved the final manuscript. 

## Financial disclosure

No financial interests related to the material of this manuscript have been declared.
